# Regulation of *CATSPER1* expression by the testis-determining gene *SRY*

**DOI:** 10.1371/journal.pone.0205744

**Published:** 2018-10-31

**Authors:** Aleida Olivares, Adriana Hernández-Reyes, Ricardo Felix, Ángela Forero, Minerva Mata-Rocha, Javier Hernández-Sánchez, Isis Santos, Charmina Aguirre-Alvarado, Norma Oviedo

**Affiliations:** 1 Unidad de Investigación Médica en Medicina Reproductiva, Hospital de Gineco- Obstetricia No. 4 Luis Castelazo Ayala, Instituto Mexicano del Seguro Social (IMSS), Ciudad de México, México; 2 Unidad de Investigación Médica en Inmunología e Infectología, Centro Médico Nacional, La Raza, IMSS, Ciudad de México, México; 3 Departamento de Biología Celular, Centro de Investigación y de Estudios Avanzados del Instituto Politécnico Nacional (Cinvestav-IPN), Ciudad de México, México; 4 Departamento de Farmacogenómica, Instituto Nacional de Medicina Genómica (INMEGEN), Ciudad de México, México; 5 Departamento de Genética y Biología Molecular, Cinvestav-IPN, Ciudad de México, México; Hokkaido Daigaku, JAPAN

## Abstract

*CATSPER1* gene encodes a pore-forming and pH-sensing subunit of the CatSper Ca^2+^- permeable channel, a protein in the flagellum essential for sperm hyperactivation. Previous studies have shown that the murine *Catsper1* gene promoter is regulated by different Sox proteins. Likewise, it is acknowledged that the human *CATSPER1* gene promoter sequence is enriched in potential interaction sites for the sex-determining region Y gene (SRY), which suggest a novel regulatory transcriptional mechanism for CatSper1 channel expression. Therefore, in this work, we sought to determine whether the human *CATSPER1* gene expression is regulated by the SRY transcription factor. To this end, a series of deletions and mutations were introduced in the wild- type *CATSPER1* gene promoter to eliminate the SRY sites, and the different constructs were tested for their ability to activate transcription in human embryonic kidney and murine spermatogonial germ cell lines (HEK-293 and GC1-spg, respectively) using luciferase assays. In addition, by using a strategy that combines electrophoretic mobility shift assays (EMSA) and chromatin immunoprecipitation (ChIP) we investigated whether the *CATSPER1* gene expression is regulated by the SRY transcription factor both *in vitro* and *in vivo*. Our results show that the transcriptional factor SRY specifically binds to different sites in the promoter sequence and has the ability to control *CATSPER1* gene transcription.

## Introduction

CatSper is a voltage-gated Ca^2+^-permeable channel specifically expressed in the sperm flagellum [1,2]. It is presumably formed by four pore-forming transmembrane subunits (CatSper1-4) [3–5], also requiring three auxiliary subunits named α, β and γ [6–8]. In comparison to other genes coding sperm Ca^2+^ channels whose disruption may not affect fertility in mouse, the expression of the *CatSper* genes seems to be essential to male fertility. Inactivation of the *CatSper* genes produces alterations in sperm hyperactivation and subsequent lower fertilization capability or infertility [6–9].

The human *CATSPER1* gene share significant homology with its murine orthologue, is located in chromosome 11 and encodes a protein of 780 amino acids with a histidine-rich domain located in the amino-terminal region [10]. This functional domain detects the changes in intracellular pH and modulates channel activity during sperm capacitation allowing a change in the movement pattern of the flagellum known as hyperactivation. *Catsper1* mRNA expression has been detected in early stages of spermatogenesis [11]. Except for *Catsper3*, which has been detected in the brain, the expression of the *CatSper* genes has been observed only in meiotic and post-meiotic sperm cells [12–14]. It has also been shown that *Catsper2* is expressed before *Catsper*1, 3 and 4, and this differential expression may be associated with different groups of genes expressed specifically in testis [14].

Likewise, it has been reported that *CATSPER1* mRNA expression is significantly lower in subfertile patients [11]; however, the causes that lead to decreased *CATSPER1* expression are presently unknown, mainly because there is limited information regarding the transcriptional regulation of its promoter and the factors that repress or activate its gene expression [15,16]. We have previously shown that the murine and human *CATSPER1* genes are driven by TATA-less promoter sequences located adjacent to the first exon. Also,we reported that the murine *Catsper1* promoter is responsive to testis transcriptional factors including Sox9 and Sox5 [17,18]. Likewise, *in silico* analysis revealed multiple sites for the sex-determining region Y gene (*SRY*) in the human *CATSPER1* promoter sequence. The *SRY* gene encodes a protein with a highly conserved DNA-binding site (79–80 amino acids), known as the HMG box that is expressed during fetal development, as well as in the adult gonadal tissue [19,20]. SRY is the transcription factor encoded by the Y chromosome, which switches on the testis determination and differentiation process(es) in the bipotential gonads. Its expression starts at embryonic day 10.5 (10.5E), and it is well known that regulates the expression of several other transcription factors including SOX9, DMRT1, GATA4, DAX1 SF1, WT1, and LHX9, and also controls the expression of diverse testicular differentiation molecules such as AMH, WNT4, FGF9, and DHH, during embryonic development [21,22].

Similar to the murine Sry, the actions of human SRY have been widely documented during testis development. However, less is known regarding its functional relevance in the adult testis. Hence, *ER71* and *SOX9* are upregulated by SRY during gonadal differentiation, and both factors regulate their expression by a transcriptional loop in the adult testis [23]. Sry regulation has also been described for the tyrosine hydroxylase *Th* gene promoter in the brain, and a role as a regulator of the Renin-Angiotensinogen system in rat and humans has also been suggested [24]. Here, we provide evidence for a novel mechanism that involves the regulation of the *CATSPER1* gene expression by SRY. Our results show that SRY may regulate either negatively or positively the *CATSPER1* gene transcription via multiple SRY binding sites located in the promoter sequence.

## Materials and methods

### Bioinformatics, luciferase reporter vectors and mutations

Potential binding sites for the SRY transcription factor within the promoter region spanning from −2153 to +102 bp of the *CATSPER1* gene were identified by MatInspector (http://www.genomatix.de/cgi-bin/matinspector_prof/mat_fam.pl) and the ConSite web server (http://consite.genereg.net/cgi-bin/consite). pCAT1, pCATΔbasal, pCATΔ3’ and pCAT739 constructs of the human *CATSPER1* promoter and *Renilla* luciferase gene were described previously [17]. A series of 5’ deletion fragments were generated from the proximal promoter construct pCATΔ3’, to eliminate the SRY binding sites, by using the QuikChange Site-Directed Mutagenesis Kit (Stratagene) and the primers used are listed in S1 Table. All primers included 25-bp from the vector sequence and 5-bp of the *CATSPER1* promoter as well as 20- to 27-bp of the desired deletions. Three deleted promoter plasmids were generated (pCATΔSRY1, pCATΔSRY2, and pCATΔSRY3) which were verified by restriction enzyme analysis and automatic sequencing. Likewise, point mutations were made for each site to prevent SRY binding to the *CATSPER1* promoter. The pCATΔ3’ was used as the template, and specific oligonucleotides (S1 Table) were used for site-directed mutagenesis using the QuikChange Site-Directed Mutagenesis Kit (Stratagene). All mutant constructs were verified by automatic sequencing.

### Construction of the pIRES-SRY vectors

The cDNA expression vector containing the sequence encoding the human SRY was initially obtained from PCR amplification of the intronless *SRY* gene using human genomic DNA as a template. According to the sequence of *SRY* obtained from GeneBank (GENE ID: 6736), the following primers were designed:

*SRY* F-5’-CACGGATCCATGCAATCATATGCTTCTGC-3’, *SRY* R-5’-TTTGAATTCC TACAGCTTTGTCCAGTGG-3’. After an initial denaturation step at 94°C for 5 min, PCR was carried out for 35 cycles at 94°C for 30 s, 58°C for 30 s, and 72°C for 1 min, followed by one step to 72°C for 10 min. A 616 bp PCR fragment was purified and subcloned into BamHI/EcoRI-digested pIREShr-GFP1a vector, and then transformed into competent *E*.*coli* DH5α cells. Positive clones were sequenced to verify the correct inserts and to obtain the pIRES-SRY vectors.

### Cell culture, transfection and luciferase assays

The human embryonic kidney (HEK-293) cell line and the murine spermatogonial germ cell line (GC1-spg) were purchased from American Type Culture Collection (ATCC). Cells were kept in culture in DMEM medium containing 10% heat-inactivated fetal bovine serum (FBS) and incubated at 37°C in a humidified incubator with 5% CO2 and 95% air. Cells were seeded at 2.5 × 10^5^ cells/well in 24-well plates for 24 h and then transfected using TurboFect transfection reagent (Dharmacon), with 1 μg of each promoter construct with deletions or 5 ng of pRL-CMV control plasmid. Fifty ng of pGL-CMV plasmid expressing *Photinus* luciferase was used as a control for transfection efficiency. Subsequent co-transfections were performed in HEK-293 cells since the mouse *Catsper1* promoter is inactive in the GC1-spg [18]. Co-transfections with mutated SRY site constructs were performed in HEK-293 cells using 500 ng of pIRES-SRY and 500 ng of each promoter construct, or pIREShr-GFP1a without SRY as a control and 25 ng of pGL- CMV. Cell lysates were prepared 48 h after transfection, and luciferase activities were determined using the Dual-Luciferase Reporter Assay (Promega). Luciferase activity was normalized with respect to the control transfection (pGL3-CMV). Data represent at least three independent experiments each performed in triplicate.

### Immunodetection of SRY in transfected cell lysates

Cell lysates were obtained from untransfected and pIRES-SRY HEK-293 transfected cells, harvested and washed with PBS and lysed in RIPA buffer. Lysates were incubated for 2 h on ice and centrifuged for 20 min at 10,000 rpm. Total protein was quantified by the Bradford method, and 15 μg were used in SDS-PAGE. Gels were transferred to PVDF membranes (Sigma-Aldrich), which were blocked and incubated with a monoclonal SRY antibody (H1; SC-374224 Santa Cruz Biotechnologies) at 1:1,000 dilution; a RNAPol II antibody (H-224; SC-9001, Santa Cruz Biotechnologies) at 1:500 dilution; a GAPDH antibody (Millipore) at 1:500 dilution, and secondary mouse or rabbit antibody (Sigma-Aldrich) at 1:15,000 dilution. Signals were revealed using a Infrared Imaging System (LI-COR Biosciences).

### Electrophoretic mobility shift assay (EMSA)

Nuclear proteins from untransfected or SRY transfected HEK-293 cells were prepared using nuclear and cytoplasmic extraction reagents (Fermentas). Protein concentration was determined using the Bradford method. Nuclear proteins were collected and stored at −80°C. The mutated and wild-type SRY site oligonucleotides used are listed S1 Table. SRY sites were called SRY3, SRY2, and SRY1 according to their localization with respect to the Transcription Start Site (TSS). Each oligonucleotide pair (5 pmol) was annealed and end-labeled with (γ-^32^P)-ATP (3,000 cpm at 10 mCi/ml) using T4 polynucleotide kinase and purified in a centrisep column. Binding reactions were carried out with 100 fmol of the end-labeled probe and 10 μgof nuclear protein SRY in binding buffer 10X (Promega), 1 μg/ml poly-dIdC, 0.05% Nonidet P-40, and 300 μg/ml BSA. Reactions were incubated at room temperature (RT) for 20 min, loaded onto 5% polyacrylamide gels and electrophoresed. For competition analysis, 2 pmol of the cold-competitor probe was included in the reaction. All experiments were performed in triplicate.

### Supershift assay with the SRY monoclonal

The wild-type SRY site oligonucleotides (S1 Table) were 3´-biotin-labeled using the Biotin 3´end DNA labeling kit (Thermo Scientific), according to the manufacturer’s protocol. Nuclear proteins were obtained from HEK293 cells as described elsewhere (Aimee Kenoyer’s non-radioactive online protocol). The binding reactions consisted of 20 fM of each labeled oligonucleotide pair; 10 μg of nuclear protein, 2μl of the IgG mouse (200 μg/ml) or monoclonal SRY antibody (200 μg/ml); 1 μg poly (dI-dC); and the aforementioned band shift buffer (Promega). The samples were incubated for 30 min at 25ºC. Negative controls consisted of 20 fM of each oligonucleotide pair and the SRY antibody, in the absence of nuclear protein. The DNA-protein complex was resolved in a Polyacrylamide gel (6%). Electrophoresis was performed in a Protean II xi system (Bio-Rad) in 0.5 TBE buffer at 10 V/cm; the gel was prerun at 15 V/cm for 20 min. Gels were transferred to nylon filters (GE Healthcare Biosciences) and cross-linking with U.V. light in a transilluminator for 5 min. Detection was performed with the Chemiluminescent Nucleic Acid detection module kit according to the instructions of the manufacturer (Thermo Fisher Scientific).

### Chromatin immmunoprecipitation (ChIP) assays

For the ChIP assays, ~1 x 10^7^ untransfected and SRY transfected HEK-293 cells, as well as immature spermatogenic cells from human semen, were used. Cells were washed in PBS and treated with 1% formaldehyde in PBS for 10 min, and 125 mM glycine for 5 min at RT. Cells were washed in PBS with a protease inhibitor cocktail (Roche) and prepared for nuclei isolation with SDS lysis buffer. Nuclear extracts were sonicated to yield DNA fragments of 200 to 900 bp. Sheared chromatin was precleared with 30 μl of protein G agarose beads overnight at 4°C. Cleared supernatant was used as input in the ChIP assay. For immunoprecipitation, crosslinked chromatin samples were added with 1 μg of each antibody for SRY (Santa Cruz Biotechnologies), RNA polymerase II (Santa Cruz Biotechnologies), a nonrelated antibody (α-dystroglycan; VIA4 Santa Cruz Biotechnologies), and mouse IgG as negative control. An aliquot of these samples was taken as input for Western blotting. Immunocomplexes were absorbed with 30 μl of protein G agarose beads for 24 h at 4°C. Samples were also taken as supernatant for Western blotting [25]. Beads were washed sequentially with low-salt, high-salt, LiCl, and TE buffer. Before elution, an aliquot of each sample was separated and boiled with Laemli buffer for Western blotting. DNA was eluted (1% SDS 0.1 m NaHCO_3_, pH 8) from beads twice, and NaCl_2_ was added overnight at 64°C to eluate input samples. After reverse cross-linking, RNAse and proteinase K was added for 1–2 h. DNA was purified by phenol/chloroform extraction followed by ethanol precipitation and resuspended in 30 μl. PCR experiments were carried out with Go Taq (Promega) using SRY0ChIP, SRY1ChIP, SRY2ChIP and SRY3ChIP oligonucleotides (S1 Table). PCR was carried out for 35 cycles by using a step cycle of 94°C for 45 s, 61°C (SRY0), 60°C (SRY1, 2), 62°C (BORIS), 63°C (SRY3) for 30 s, 72°C for 30 s, followed by a step to 72°C for 10 min. The input was diluted (1:10) and 1 μl of DNA from each immunoprecipitation assay was directly used for PCR assays. Products of SRY sites of 244, 348, 137 and 248 bp were analyzed by electrophoresis on a 2% agarose gel. A 300 bp BORIS promoter region without SRY sites and rich in GC was amplified as a negative control. All experiments were performed in triplicate. Western blot analyses were performed to validate SRY binding to the agarose beads during chromatin immunoprecipitation. Samples from the input, supernatant, and protein bound to beads (output) were boiled with Laemli buffer, separated in SDS-PAGE using the conditions above mentioned for SRY Western blot.

## Results

### SRY binds to specific site in the CATSPER1 promoter

**Fig 1:**
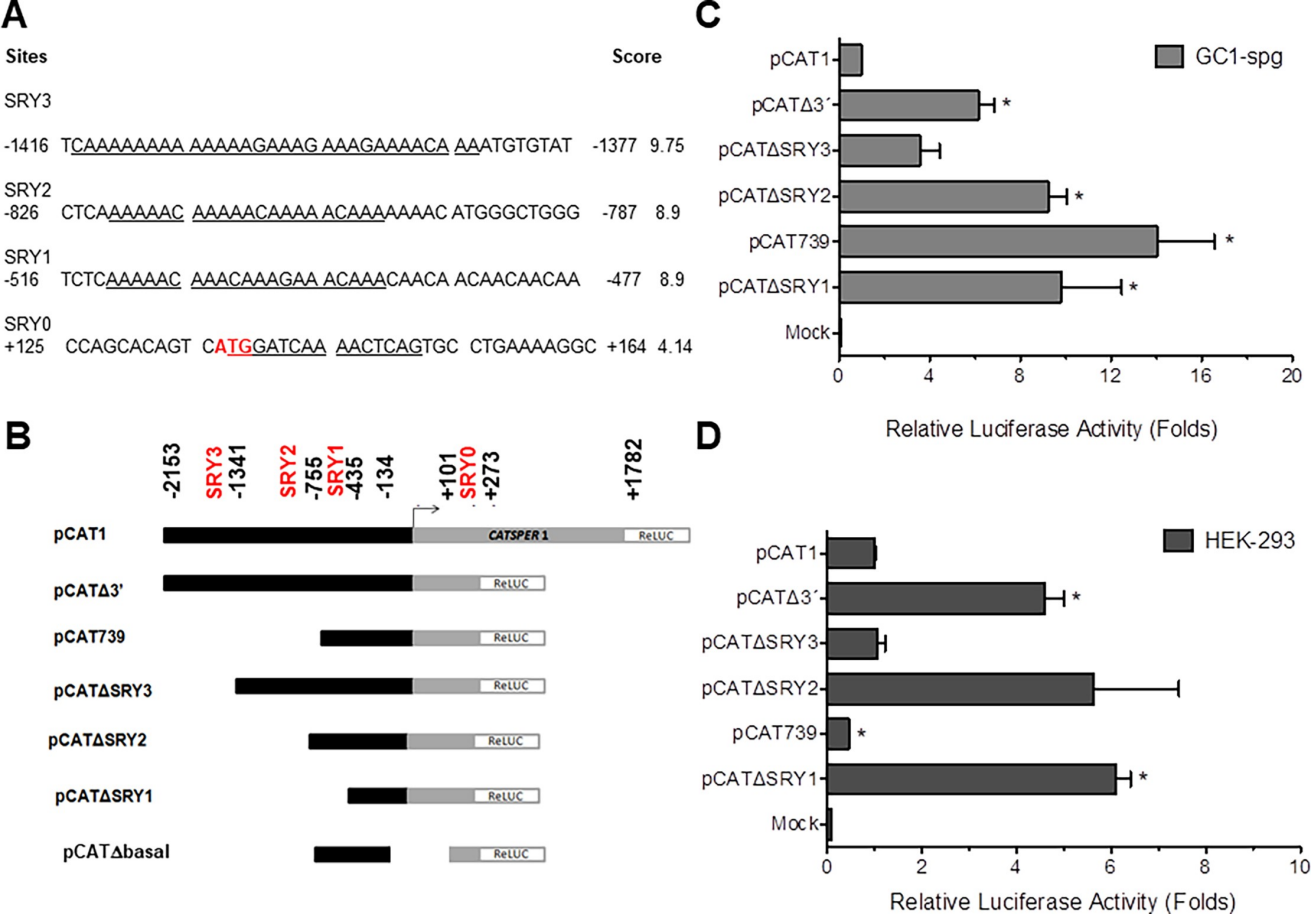
Identification of SRY sites in the *CATSPER1* gene promoter. A) Four SRY sites were identified *in silico* by MatInspector and ConSite software. Tandem and single SRY sites are underlined, numbers correspond to upstream position (negative numbers) or downstream position (positive numbers) relative to the TSS of the *CATSPER1* gene. B) *CATSPER1* promoter deletion constructs and localization of the SRY sites. The *CATSPER1* promoter was cloned in the pRL vector (Promega), and a series of sequential deletions that eliminate the first exon and upstream regions were designed to target SRY sites. These constructions were transfected in two cell lines, GC1-spg (C) and HEK-293 (D). Luciferase activities were measured and the relative activities were normalized with respect to the total activity from the pCAT1 construct. Results are presented as mean ± SEM (n = 3). pRLnull was used as negative control in mock-transfected cells. Statistically significant differences from control levels (pCAT1) are indicated by *; P<0.05.

We have previously reported that diverse *CatSper1* constructs, including one called pCATΔ3’, show significant promoter activity [17,18]. Here, by using *in silico* analysis we have detected three overlapping SRY binding sites in this region termed SRY1 (-511/-491), SRY2 (-822/-802) and SRY3 (-1405/-1386), as well as a single SRY site in a downstream region called SRY0 (+137/+145; Fig 1A). Therefore, a set of pRL-derived constructs containing different fragments of the 5’ region of the *CATSPER1* gene promoter upstream of the *Renilla* luciferase reporter were prepared and evaluated in the GC1-spg and HEK- 293 cell lines. All deletion constructs were generated to eliminate either the overlapping SRY sites or the single SRY site from the *CATSPER1* promoter region using the pCATΔ3’construct as a template (Fig 1B).

When expressed in GC1-spg cells, the plasmid pCAT739 showed the highest relative activity (>14-fold), while deletions of 1718 and 1398 bp, respectively, in the pCATΔSRY1 and ΔSRY2 constructs also showed a significant increase in activity (~10-fold) in relation to pCATΔ3’, the construct that contained all the SRY binding sites (Fig 1C). This construct showed ~6-fold increase in luciferase activity relative to the full-length construct pCAT1. The ΔSRY3 deletion plasmid showed lower transcriptional activity (~4-fold) in comparison to pCAT1 but displayed less luciferase activity that pCATΔ3’ (Fig 1C). In contrast, when expressed in HEK-293 cells, the transcriptional activity of pCATΔSRY1 and ΔSRY2 were similar (~6-fold) to that displayed by pCATΔ3’ (5-fold), but the pCATΔSRY3 construct showed decreased basal activity (~1.4-fold) in comparison to the activity observed when the pCATΔ3’ was transfected (Fig 1D). It is worth noting that the relative activity in the mock-transfected GC1sp cells is twice compared to the HEK-293 cell line, due possibly to the presence of endogenous Sox factors in the spermatogonial cell line. Together, these results suggest that the elimination of regions containing SRY binding sites affect the transcriptional activity of the *CATSPER1* gene promoter.

It should be clarified also that the expression of *CATSPER1* is not detectable in HEK-293 cells even after SRY overexpression. In addition, expression of *CatsSper1* was not detected in the GC1spg cell line though these cells are derived from spermatogonia. Despite the fact that the promoter is specifically activated in sperm under physiological conditions, our data suggest that transcriptional activity in transfected cells is twice as high in GC1spg as in HEK-293 cells, and in both cases, this activity is higher than in the mock condition (S1 Fig). It is also worth mentioning that the episomal plasmids transfected into HEK-293 cells also responded to the overexpression of the human SRY factor, and it is possible that the GC1sp cells express other Sox factors contributing to the exacerbated activity observed.

### SRY modifies the CATSPER1 promoter transcription

**Fig 2:**
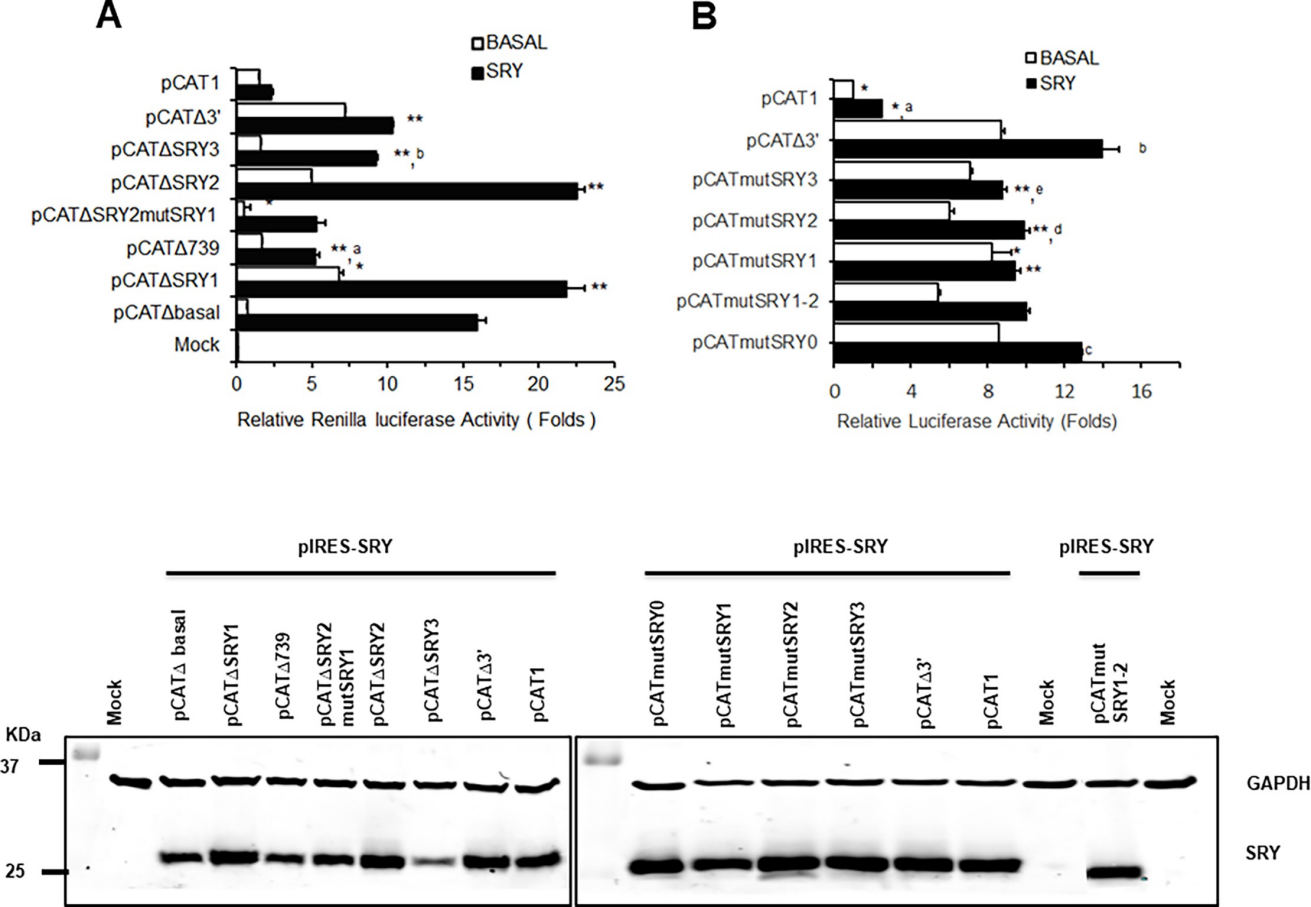
SRY regulates the *CATSPER1* promoter. A) Constructs of the *CATSPER1* promoter, as in Fig 1B were transfected in HEK-293 cells alone or in conjunction with the pIRES-SRY vectors as indicated. The basal transcriptional activity of the *CATSPER1* promoter (open bars) and the transcriptional activity of the *CATSPER1* promoter after pIRES-SRY overexpression (closed bars) are plotted. The relative luciferase activity was normalized to the activity obtained with pCAT1. Results are presented as mean ± SEM (n = 6). pIRES hr-GFP1a vector was used as negative control. B) Comparison of constructs harboring point mutations for the SRY sites generated from pCATΔ3’. Constructs with the mutations in the SRY sites and the controls were transfected in HEK-293 cells and luciferase activities were measured. The basal transcriptional activity of the promoter (open bars) and the transcriptional activity after overexpression of SRY in mutated constructs (closed bars) are plotted. The relative luciferase activity was normalized with respect to the basal activity obtained from the pCAT1 construct. In all cases, statistically significant differences among conditions are indicated by *, P<0.05. Letters indicate differences in transcription levels between basal and SRY overexpressing cells, P<0.05. The lower panels show the immunodetection of SRY and GAPDH proteins from lysates of pIRES-SRY co-transfected cells for each condition of observed transcriptional activity (mock corresponds to transfected cells with pIRES empty vector).

To explore the role of the SRY transcription factor on *CATSPER1* expression, we next evaluated the effect of SRY overexpression. To this end, some of the above described *CATSPER1* promoter constructs were co-transfected with an SRY plasmid (pIRES-SRY), and their basal and induced transcriptional activities determined by luciferase assays after 48 h of transfection (Fig 2A). The results of this analysis show an increase in the relative luciferase activity in transfected cells expressing the pCATΔbasal, pCATΔ3’ and pCAT739 constructs when the plasmid encoding SRY was co-expressed. Indeed, transcription activity of pCAT739 and pCATΔbasal plasmids was induced ~4- and ~10-fold with SRY, while pCATΔ3’ luciferase activity augmented ~8-fold relative to pCAT1.

Given that these plasmids represent large regions in the promoter sequence, our results suggested a potential regulation through SRY but does not indicate necessarily whether the transcription factor occupies the conserved binding sites found in the *in silico* analysis. For this reason, we decided to use constructions in which SRY binding sites would be eliminated so that they could be analyzed more easily. Thus, deletions in pCATΔSRY3 and pCATΔbasal showed an increase of >10–15 fold, pCATΔSRY1 and pCATΔSRY2 showed a >20-fold increase with respect to the pCAT1 construct (Fig 2A). Deletion of the SRY2 site and mutation of the SRY1 site on the *CATSPER1* promoter render the lowest transcriptional activity and diminish 15 fold its activity with the SRY overexpression (at the same level than pCATΔ739). These results suggest a regulatory role for the SRY transcription factor on the *CATSPER1* promoter activity mediated by the three predicted binding sites.

### Mutational analysis of the SRY binding sites

In order to corroborate the functional role of the SRY binding sites in the *CATSPER1* promoter, we next introduced mutations in the four identified binding sites. As mentioned earlier, three of these sites are located upstream of the TSS (SRY1-3), and one site (SRY0) is located downstream of the TSS and the start codon. Bioinformatic analysis was performed to verify that the mutant sequences did not introduce possible new SRY sites or any other site for additional transcription factors. Mutations were made using the pCATΔ3’ construct as a template by PCR site-directed mutagenesis and verified by automatic sequencing.

Basal transcription activity was evaluated and compared with the wild-type (WT) *CATSPER1* promoter (pCATΔ3’). We used pCAT1 basal activity to normalize data. Our results show small changes (~20%) in the relative activity of pCATmutSRY3 and pCATmutSRY1 mutant construct with respect to the WT pCAT1Δ3’ construct (Fig 2B). Only pCATmutSRY2 and the double mutant SRY1-2 showed decreased basal activity, which was restored after SRY overexpression. In contrast, co-transfection of pCATmutSRY3 and pCATmutSRY1 with pIRES-SRY in HEK-293 cells significantly decreased transcription with respect to the WT pCAT1Δ3’ construct (Fig 2B). Likewise, co-expression of SRY and the pCATmutSRY0 construction resulted in a significant increase (~50%) in luciferase activity.

### Nuclear proteins binding to the SRY2 site

**Fig 3:**
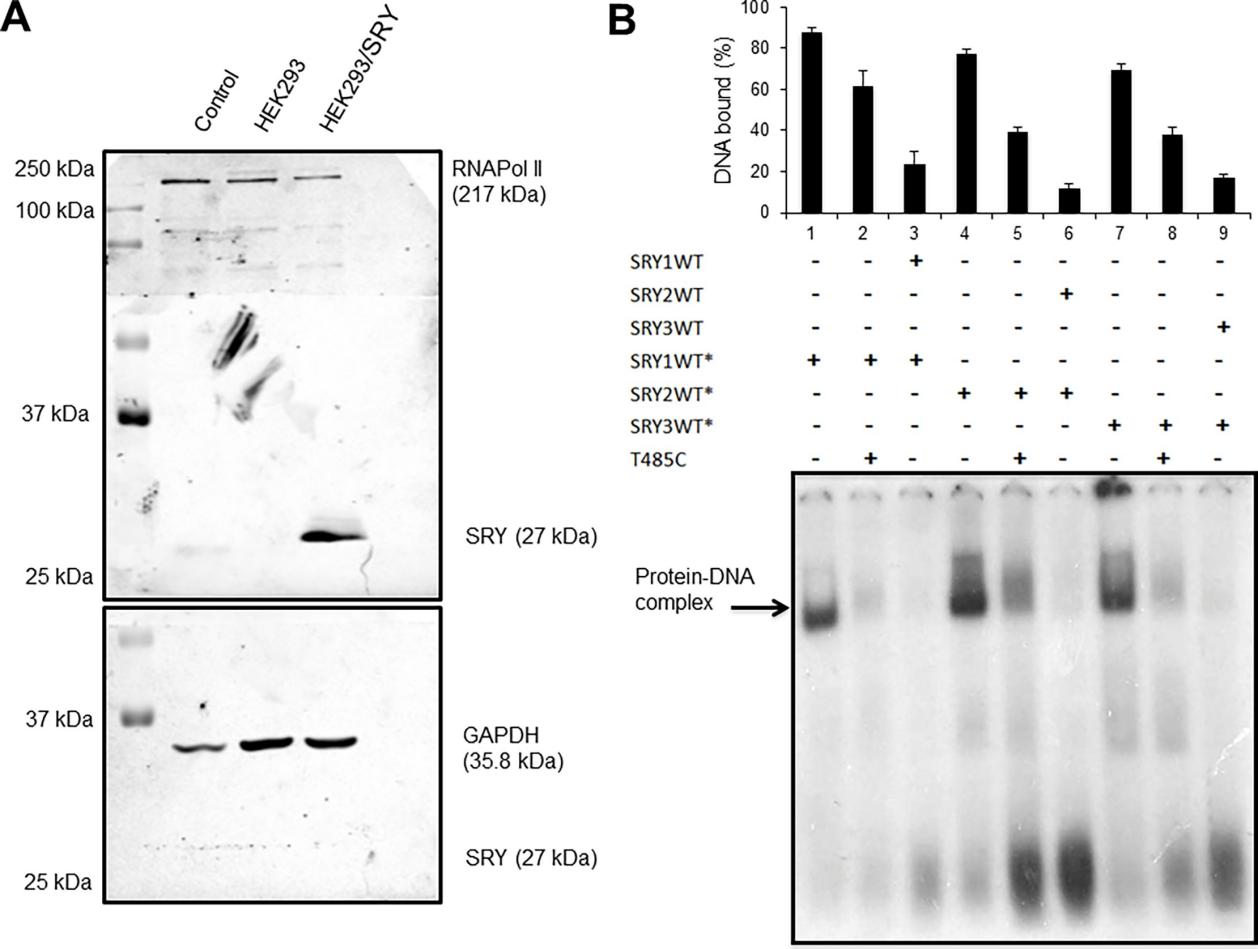
Nuclear protein binding to SRY sites *in vitro*. A) The upper panel shows the expression of SRY and RNAPol II, respectively, in nuclear protein extracts from untransfected (line HEK293), pIRES SRY-transfected HEK-293 cells (HEK293/SRY) and 112-D cells as a negative control (Control); SRY antibody was also assayed in cytoplasmic extracts and GAPDH was used as load control in (lower panel). B) EMSA experiments showing the complex formation among labeled SRY1WT*, SRY2WT* and SRY3WT* sites (1 pmol) in the presence of 10 μg nuclear proteins from SRY transfected HEK-293 cells (lines 1, 4, 7). For the competition assay, 2 pmol of unlabeled WT oligonucleotides (SRY1WT, SRY2WT, and SRY3WT in lines 3, 6 and 9) and nonspecific oligonucleotides (T485C in lines 2, 5 and 8) were added to the reaction mixture, before the addition of labeled probes. The upper panel shows the quantification of the DNA bound in each line.

To investigate the potential binding of nuclear proteins to the SRY sites, we next performed electrophoretic mobility shift assays (EMSA). SRY sites were represented in duplex oligonucleotides as a WT and mutated versions. The overexpression of SRY and its nuclear location was corroborated by Western blot of SRY in nuclear and cytoplasmic extracts from pIRES-SRY transfected HEK-293 cells (Fig 3A). EMSA assays were performed using ^32^P-labeled oligonucleotide probes for the WT SRY sites. Initially, nuclear proteins from SRY-transfected HEK-293 cells were tested using ^32^P-labeled and unlabeled duplex WT oligonucleotides under binding conditions. Each of the three upstream SRY sites (SRY1-3) showed the formation of a covalent DNA-protein complex after incubation of the probes with the nuclear extract obtained from pIRES-SRY transfected cells. Such DNA-protein complexes diminished when an excess of non-specific duplex oligonucleotides was added to the reaction, suggesting that other nuclear proteins are bound to these oligonucleotides in an unspecific way. However, the complex formation was abolished in the presence of a specific unlabeled competitor oligonucleotide (100-fold molar excess; Fig 3B).

**Fig 4:**
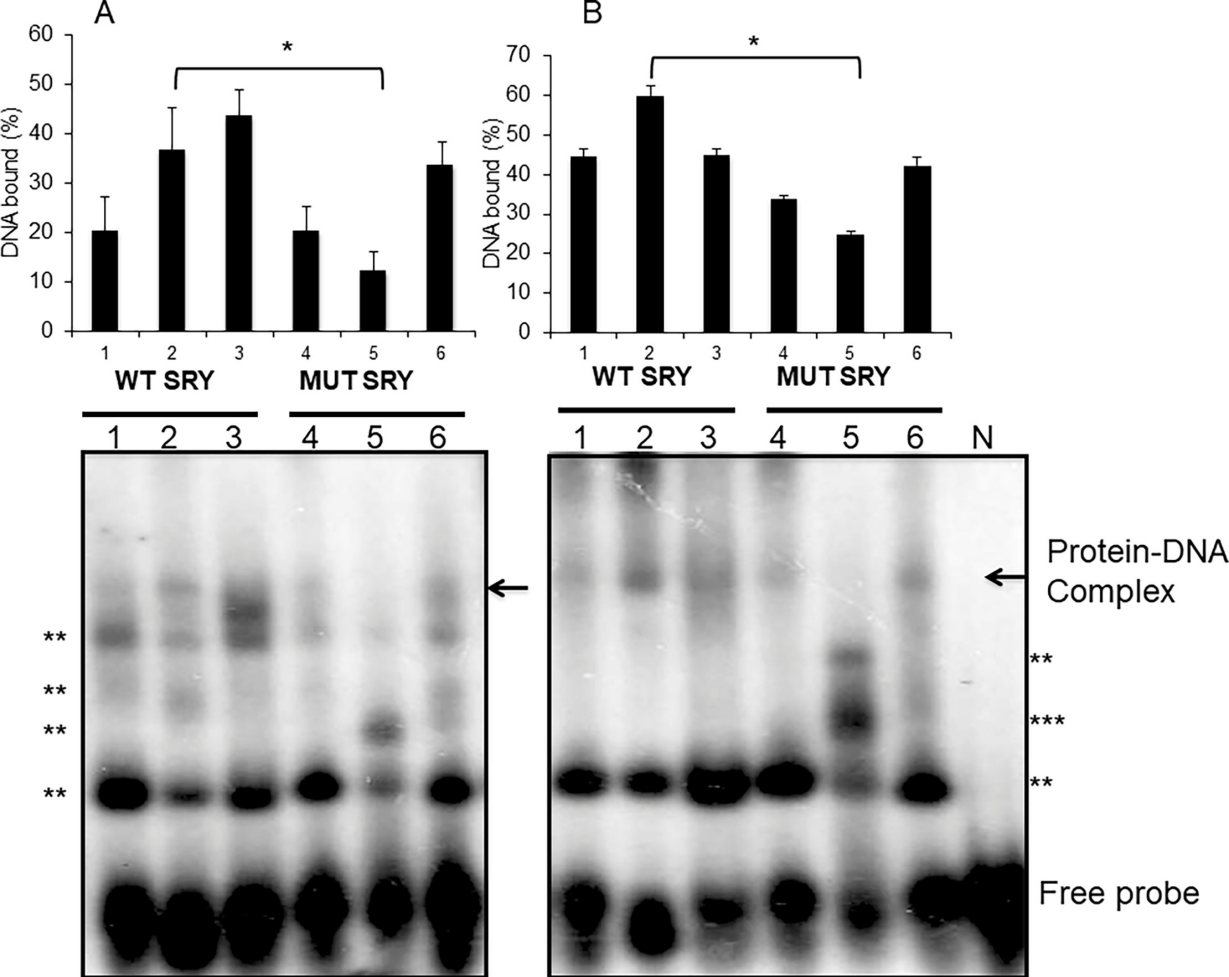
Mutations in SRY sites and their binding assays with nuclear proteins A) EMSA experiments of SRY probes with nuclear proteins from untransfected HEK-293 cells. Lines 1, 2 and 3 contain labeled WT SRY sites (1–3, respectively), lines 4, 5 and 6 correspond to labeled mutated SRY sites (1–3, respectively) with 10 μg nuclear proteins. B) EMSA of SRY sites WT (lines 1–3) and mutated (lines 4–6) with nuclear proteins from SRY transfected HEK-293 cells. SRY1 labeled probe without nuclear protein was used as negative control (NC). A representative experiment from three independent experiments is shown. Statistically significant differences between wild-type and the mutant constructs are indicated by *; P<0.05,** indicates non-specific bands and *** indicates a remaining band after SRY overexpression.

The mutant duplex oligonucleotides were then tested for their ability to bind nuclear proteins from pIRES-SRY expressing cells. As expected, the WT oligonucleotides formed DNA-protein complexes, however, the presence of the three mutations in SRY2 abrogated the formation of such complexes suggesting that only SRY2 site specifically binds SRY *in vitro*. Though the other mutant oligonucleotides eliminated two of the SRY1 and SRY3 sites, DNA-protein complexes were still observed given that other weak binding sites might still be recognized by SRY or by other Sox factors (Fig 4A and 4B).

**Fig 5:**
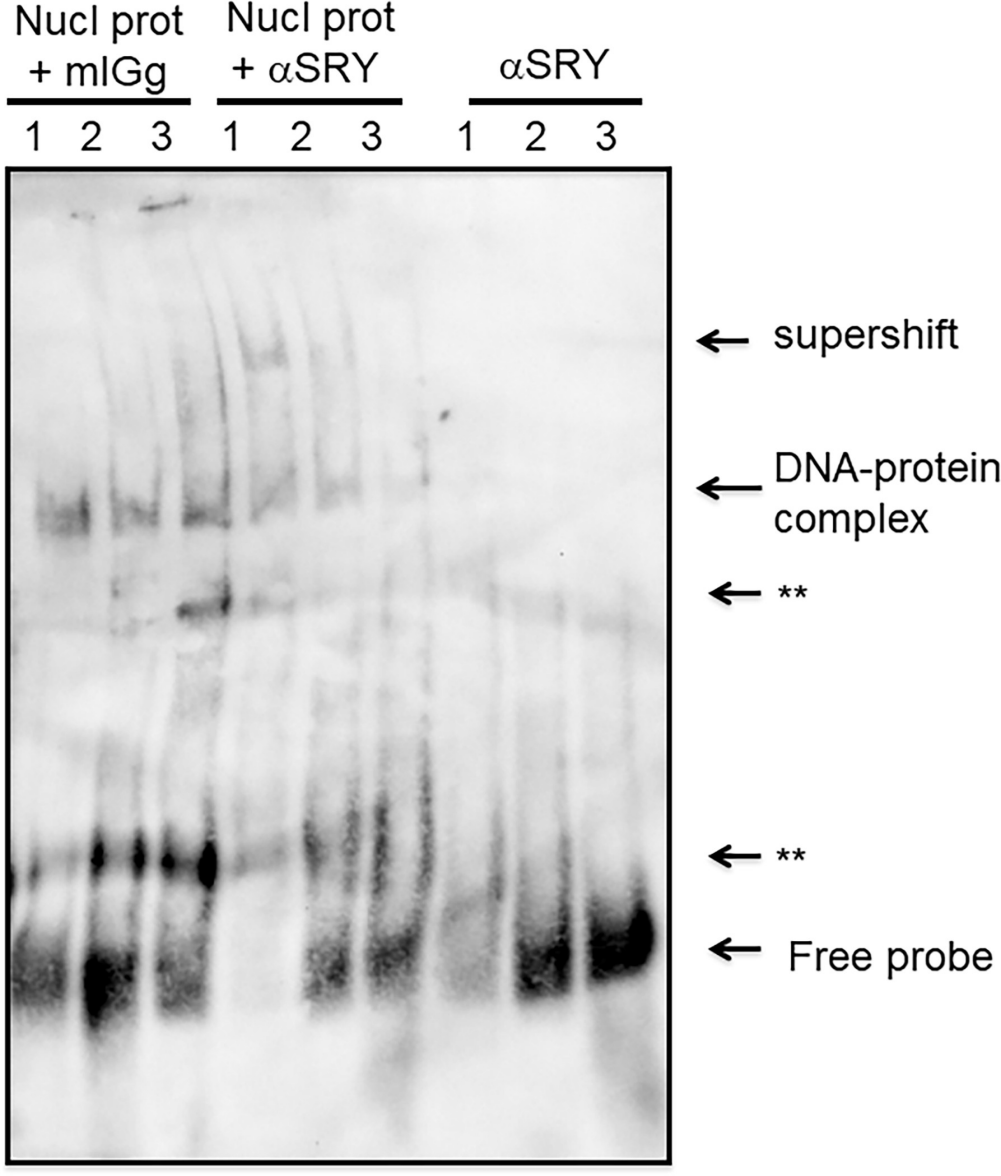
Supershift of SRY1 and 2 sites with the SRY antibody. Experiments were performed with biotin labeled probes in the same conditions as EMSA assays previously performed, blotted to nylon membranes and revealed with Streptavidin-HRP by chemiluminescence detection. Lines 1, 2 and 3 contain biotin-labeled WT SRY sites (1–3, respectively), with nuclear proteins from SRY transfected HEK293 cells and IgG mouse, and the same probes with nuclear proteins plus 0.4 μg of monoclonal SRY antibody (αSRY). Arrows indicate the DNA-protein complexes and the supershift in the SRY1 and SRY2 sites. The asterisks indicate non-specific bands, and the negative control of unspecific binding of the SRY antibody with biotin-labeled probes (without nuclear proteins) is shown in the three lanes at the right.

In order to identify a specific interaction with SRY, supershift assays were performed with a monoclonal SRY antibody and biotin 3´labeled oligonucleotides with SRY sites. The results show a signal for the specific interaction of the SRY antibody with the nuclear protein bound to the SRY 1 and SRY2 sites (Fig 5).

### SRY recognizes the SRY binding sites in the CATSPER1 promoter

**Fig 6:**
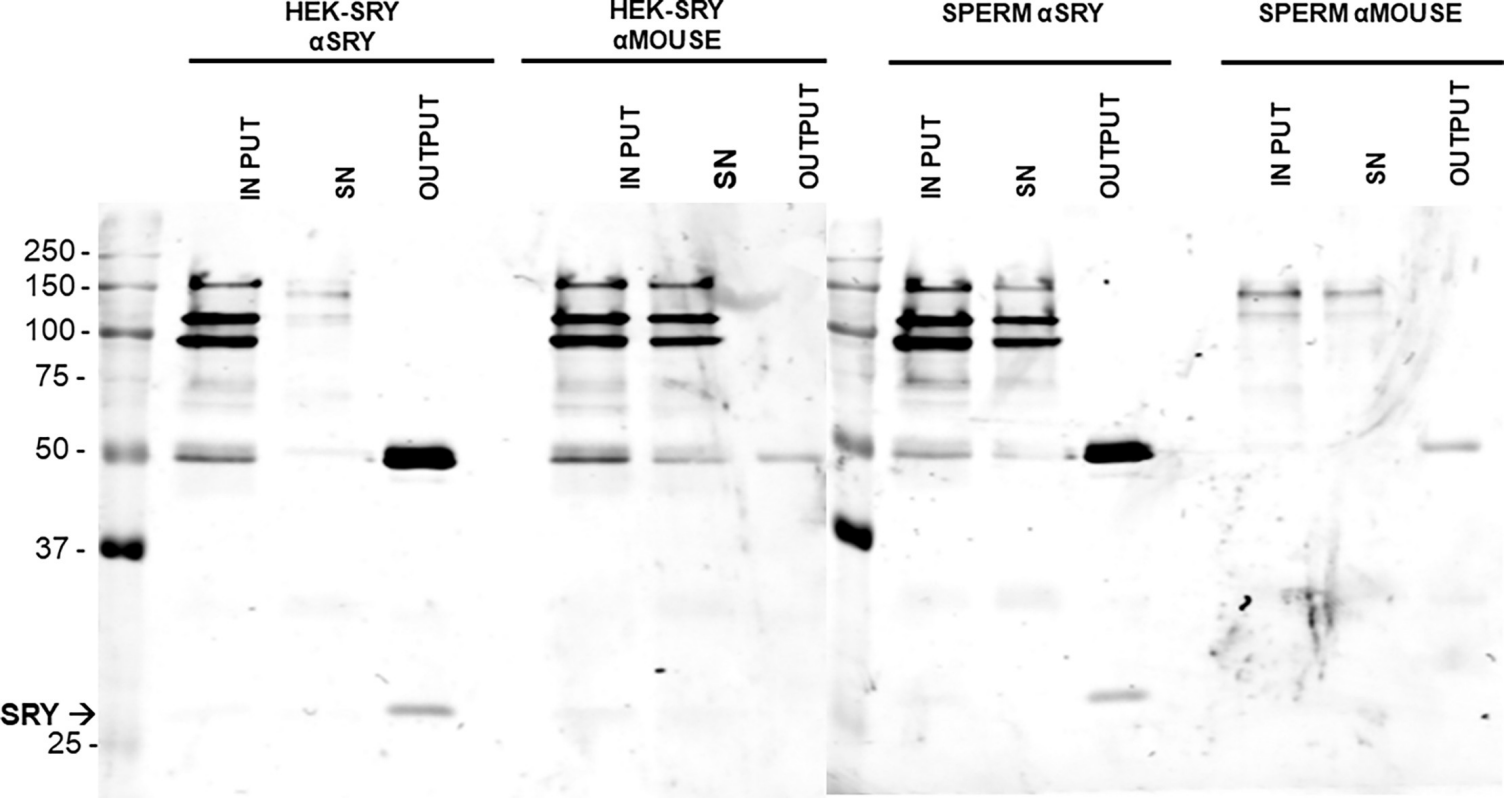
SRY binding to SRY0 and SRY2 sites in the *CATSPER1* promoter. Immunoblotting for SRY in samples from ChIP assays. Chromatin from pIRES-SRY transfected HEK-293 cells (HEK-SRY) and immature spermatogenic cells from human semen (SPERM) was probed in immunoprecipitation assays using anti-mouse IgG, SRY (α-SRY) and α-dystroglycan (α-MOUSE) antibodies. A sample of 2.5% of input, 2.5% of supernatant (SN) and 10% of bound protein to beads (output) was immunodetected with the SRY specific antibody in each condition. The arrow indicates the SRY signal.

**Fig 7:**
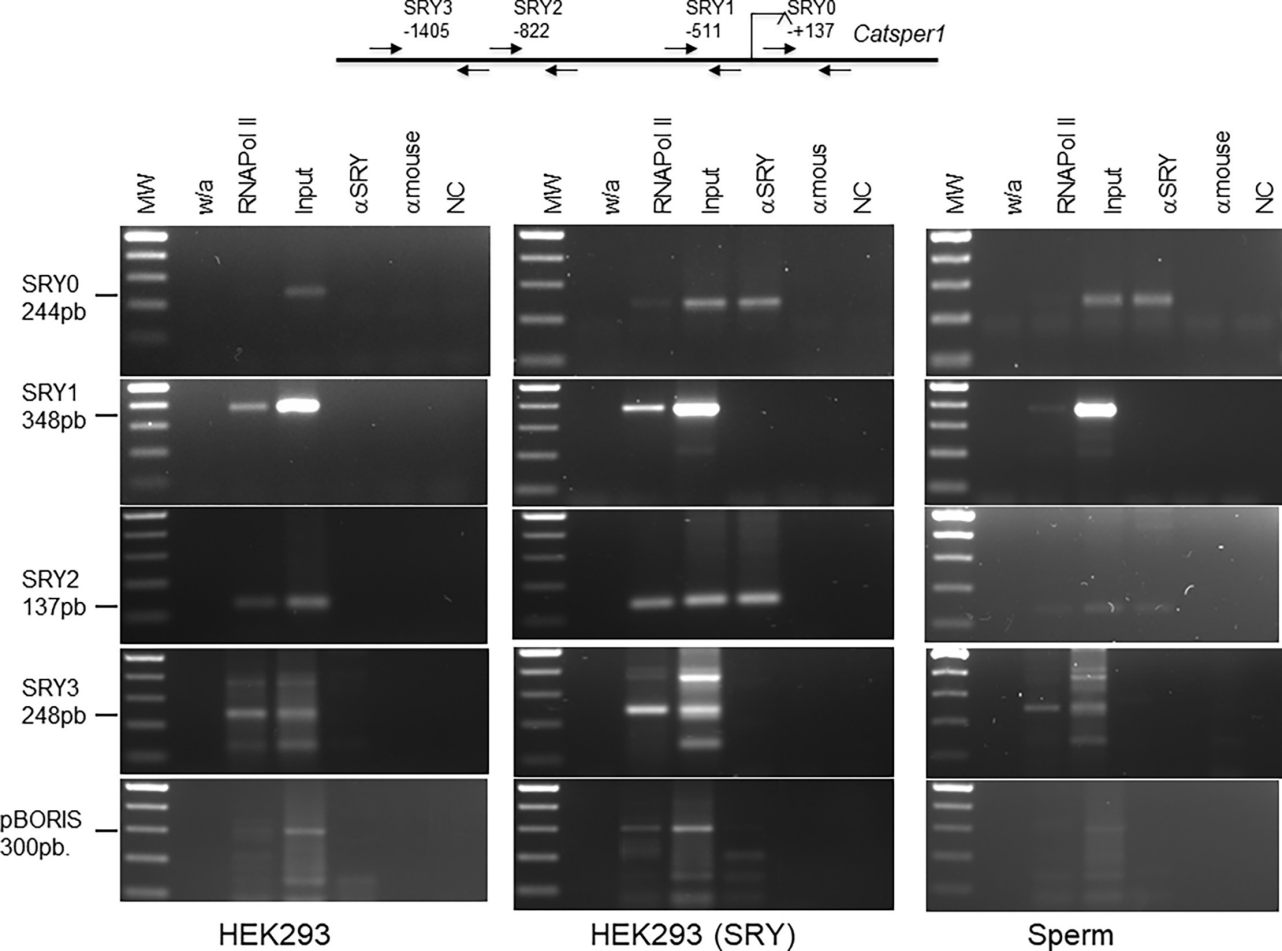
ChIP assay of the *CATSPER1* promoter at the SRY overlapping binding sites. Immunoprecipitation of chromatin fragments was carried out with 1 μg of RNA polymerase II (RNAPol II), and SRY (α-SRY) and α-dystroglycan (α-mouse) antibodies in untransfected and pIRES-SRY transfected HEK-293 cells as well as in spermatogenic cells. Chromatin without antibody, the IgG mouse and the α-dystroglycan antibody were used as negative controls; RNAPol II and 1/10 of Input were used as positive controls. After reverse cross-linking, RNAse and proteinase K treatments were performed and the purified DNA samples were analyzed by PCR with oligonucleotides for each SRY site. As a negative control for PCR, water was added instead of the immunoprecipitated chromatin fragments (NC). A fragment from the BORIS promoter (GC-rich) was amplified as a negative control (unspecific SRY binding). Representative PCR amplifications for the different SRY sites are shown (lower panel; n = 3).

The potential *in vivo* interaction of the SRY transcription factor with the *CATSPER1* promoter via their specific binding sites was next explored using ChIP assays. DNA samples were immunoprecipitated from HEK-293 and HEK-293 (SRY) cells as well as human ejaculates using SRY antibodies. Immunoprecipitation of SRY during ChIP was verified by Western blot analysis from chromatin samples of HEK-293 (SRY) and sperm cells (Fig 6). SRY was detected with a specific SRY antibody but not with a non-related antibody (mouse IgG). Likewise, DNA fragments of 244, 348, 137, and 248 bp were amplified from the *CATSPER1* promoter (Fig 7). Strong signals for SRY2 and SRY0 were observed in HEK-293 (SRY) and sperm using their respective control isotype (α-mouse), while SRY1 and SRY3 signals were negative in both cell types. In contrast, any SRY site was amplified in untransfected HEK-293 cells, thought all SRY sites were amplified in the α-RNAPol II immunoprecipitation assay (Fig 7). These results suggest that the transcription factor SRY may bind to the specific SRY2 and SRY0 sites *in vivo*, and corroborate that SRY2 is an important regulation site in the human *CATSPER1* gene promoter.

## Discussion

This work provides what is to our knowledge the first evidence concerning the regulation of the *CATSPER1* gene promoter by the HGM-box transcription factor SRY. *CATSPER1* encodes a plasma membrane ion channel required for sperm hyperactivation and male fertility [10]. Previous studies have shown that *CatSper1* is exclusively expressed in human and mouse sperm [1,2]. Despite extensive characterization of the functional importance of human *CATSPER1* gene, little is known regarding the transcriptional factors that regulate its expression.

The murine *Catsper1* promoter is responsive to Sox5 and Sox9 transcription factors [18]. However, no binding sites for Sox proteins have been identified in the human *CATSPER1* gene promoter. Instead, the *CATSPER1* promoter comprises several binding sites for the Y-linked sex-determining gene (SRY). Interestingly, Sox proteins and SRY belong to the same family of HMG-box proteins, and their consensus binding sites contain the same core (AACAA) with minimal differences of one or two bases at the end of the sequence. However, it is possible that different Sox factors may recognize the same binding site in the promoter, and then the differential expression of Sox factors according to the cell type becomes relevant as well as the recruitment of other factors to the promoter.

The *CATSPER1* promoter contains three overlapping SRY sites upstream the TSS (SRY1-3) and one single SRY site downstream the TSS (SRY0). Deletion analysis of the promoter shows a loss of transcriptional activity upon removal of the region containing the SRY3 site, which is recovered when the region containing the SRY2 site is removed. Since both regions are extensive, these findings could be the result of a loss of sites for other transcription factors. However, a mutation in the SRY1 site introduced into the pCATΔSRY2 construct virtually eliminates basal transcriptional activity. On the other hand, SRY overexpression increases transcriptional activity in all constructs, since they contain at least one of the SRY sites. Indeed, we found that mutation of SRY2 had a decreasing effect on basal transcriptional activity when the other SRY sites remained intact in the promoter sequence, even with the mutated SRY1 site in the double mutant pCATmutSRY1-2. This decrease in activity was prevented after SRY overexpression. On the other hand, the SRY mutants did not reach the same level of transactivation by SRY overexpression with exception of SRY0 mutant. Last, EMSA, supershift and ChIP analysis revealed a specific interaction between SRY and the SRY2 site. These findings imply that SRY2 is an important site for the regulation of the *CATSPER1* gene promoter activity.

Interestingly, three SNPs have been detected in the SRY2 site, called rs7129846, rs201387881, and rs200309988. These SNPs are present in the core of the SRY binding site and correspond to a C↔A change, an AC/A deletion and a C/CA insertion, respectively. The rs7129846 is observed with an allele frequency of C = 0.08 A = 0.91 in the Mexican ancestry from Los Angeles, CA, USA, (https://www.ncbi.nlm.nih.gov/variation/tools/1000genomes/; August 2017) [26]. Thus, it is possible that these differences in the *CATSPER1* promoter sequence may determine a differential expression pattern among individuals. In this context, it is worth noting that low expression of *CATSPER1* is related to impaired fertility because of reduced sperm mobility [11,13,27]. Although differences in *CATSPER1* expression may occur in populations of sperm with normal and altered motility [13,27], the SNPs in the SRY2 site might help explain why some individuals that show reduced levels of *CATSPER1* expression also present poor sperm motility. Likewise, the presence of overlapping SRY sites in the *CATSPER1* promoter might help to prevent the negative effects of the mutations or SNPs on *CATSPER1* expression, ensuring gene expression and preventing alterations in sperm motility.

It is worth noting here that ENCODE and roadmap epigenomics data suggest multiple transcription factors binding (DNase and ChIP-Seq), histone modification (H3K27Ac) of an active state in nonexpressing cells near exon 2 of the gene, and a general open chromatin state for the entire gene. Yet there is no expression in these cells. These data suggest the presence of much more complex patterns of promoter regulation.

In addition, SRY may serve as an important transcriptional factor to regulate the tissue-specific expression of *CATSPER1* during spermatogenesis. Sry expression has been documented in different tissues such as kidney, adrenal gland, and brain, as well as in the adult testis [28,29]. Likewise, the *SRY* gene promoter contains multiple sites for the transcription factor Sp1. Likewise, the *Sry* promoter lacks the TATA box sequence, and experimental data suggest a differential *SRY* transcription that depends on tissue- and species-specific factors. *In vitro* studies during gonadal differentiation have shown that transcription factors WT1, NR5A1, Sox9, Gata4, and Sp1 may bind to the promoter and activate *Sry* gene transcription. Similarly, it has been shown that during *Sry* expression, the levels of a histone demethylase (H3K9me2) are reduced favoring transcription initiation. Last, it has been reported that an isoform of the WT1 protein (Wt1+ KTS) contributes to the posttranscriptional regulation of the *Sry* messenger RNA [24].

In summary, here we show that SRY may regulate *CATSPER1* gene transcription via multiple SRY binding sites located in the promoter sequence. This is likely to assist in defining the key factors responsible for physiological and pathophysiological CatSper expression. Given the proposed importance of this protein in fertilization, identification of the mechanisms controlling its expression could also be of clinical use. Furthermore, since variations in the sequence of the SRY2 site have recently been shown to give rise to individual variability of *CATSPER1* expression, knowledge of the promoter sequence and regulation might eventually help to ensure gene expression and prevent alterations in sperm motility.

## Supporting information

S1 Fig Comparison of the pCAT1 construct transcriptional activity in HEK-293 and GC1-spg cells. (TIF)

S1 Table (DOCX)
