# Supplementary figures and images for: Regulation of CATSPER1 expression by the testis-determining gene SRY
Source: PLoS One. 2018 Oct 31;13(10):e0205744. doi: 10.1371/journal.pone.0205744 (PMC6209213; doi:10.1371/journal.pone.0205744)

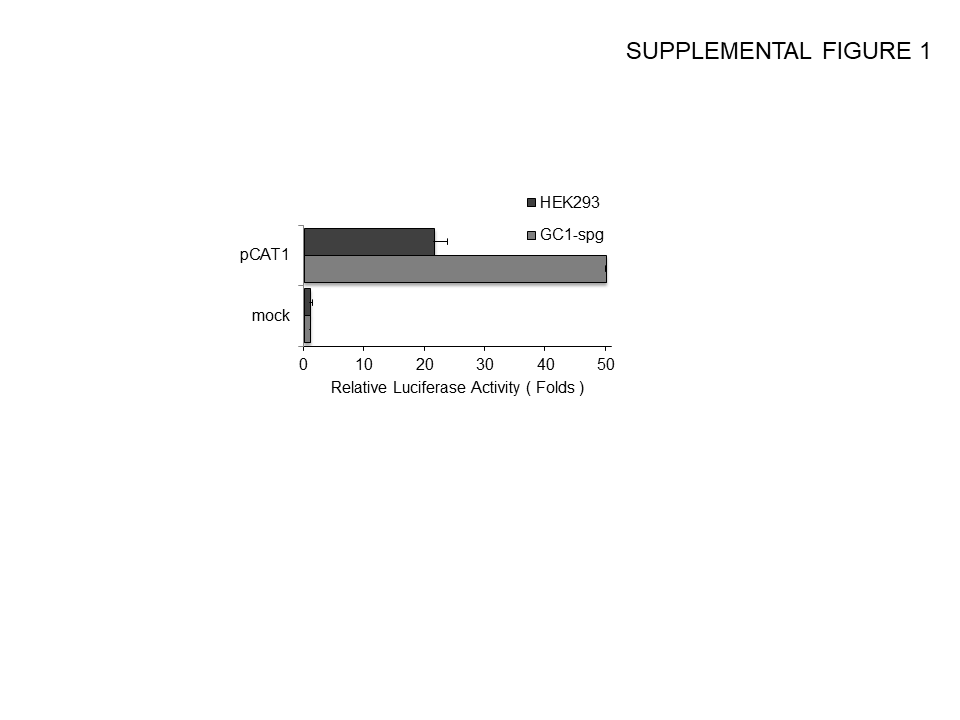

Supplement: S1 Fig — (TIF) [file pone.0205744.s001.TIF]
