# Supplementary material for: Regulation of CATSPER1 expression by the testis-determining gene SRY
Source: PLoS One. 2018 Oct 31;13(10):e0205744. doi: 10.1371/journal.pone.0205744 (PMC6209213; doi:10.1371/journal.pone.0205744)
Supplement: S1 Table — (DOCX) [file pone.0205744.s002.docx]

| PRIMER | SEQUENCE | USE |
| --- | --- | --- |
| ΔSRY1 Forward | 5′- CGAGCTCTAAGCTTACTGGGAACCCCATGTATTATGCTGTTAATATAAGGC-3' | Deletion |
| ΔSRY1 Reverse | 5′- GCCTTATATTAACAGCATAATACATGGGGTTCCCAGTAAGCTTAGAGCTCG-3' | Deletion |
| ΔSRY2 Forward | 5′-CGAGCTCTAAGCTTACTGGGAACCCTGGGCGCAGTGGCTCACAC-3' | Deletion |
| ΔSRY2 Reverse | 5′-CGAGCTCTAAGCTTACTGGGAACCCTGGGCGCAGTGGCTCACAC-3' | Deletion |
| ΔSRY3 Forward | 5′-GAGCTCTAAGCTTACTGGGAACCGAAGCAATCCTCAATAAATGATGG-3' | Deletion |
| ΔSRY3 Reverse | 5´-CCATCATTTATTGAGGATTGCTTCGGTTCCCAGTAAGCTTAGAGCTCG-3' | Deletion |
| SRY0CHIPF | 5’-CCATCTCAAAAACAAACAAAGAAAC-3’ | ChIP |
| SRY0CHIPR | 5´-GCCTTATATTAACAGCATAATACATG-3´ | ChIP |
| SRY1CHIPF | 5´-GAGCTTGGCTCAGGAAAGAAG-3’ | ChIP |
| SRY1CHIPR | 5’-GAGCCTGGCCTGTGGTGTGGGGG-3’ | ChIP |
| SRY2CHIPF | 5’-AGA TTG CCA TGA GTG GAG ATC-3’ | ChIP |
| SRY2CHIPR | 5’-CTT CCA AAG TGT TGG GAT TAC A-3’ | ChIP |
| SRY3CHIPF | 5’-TTC AGG AGG TGG AGG TTG CT-3’ | ChIP |
| SRY3CHIPR | 5’-TTC AGG TGT GAG CCA CCA TG-3’ | ChIP |
| SRY3MUTFOR  SRY3MUTREV | 5’-CTGTGTCTCAAAAAAAAAAAAAG**CCG**GAAAGA**GGG**CAAAATGTGTATGAAGCAATCCTC-3’  5´-GAGGATTGCTTCATACACATTTTGCCCTCTTTCCGGCTTTTTTTTTTTTTGAGACACAG-3´ | EMSA  Mutagenesis |
| SRY3WTFOR  SRY3WTREV | 5’-CTGTGTCTCAAAAAAAAAAAAAG**AAA**GAAAGA**AAA**CAAAATGTGTATGAAGCAATCCTC-3’  5´-GAGGATTGCTTCATACACATTTTGTTTTCTTTCTTTCTTTTTTTTTTTTTGAGACACAG-3´ | EMSA  Mutagenesis |
| SRY2MUTFOR  SRY2MUTREV | 5’-GACAGTGAGACTGTCTCAAA**GGG**CAA**GGG**CAAAAACA**GGG**AAACATGGGCTGGGCGCAG-3’  5´-CTGCGCCCAGCCCATGTTTCCCTGTTTTTGCCCTTGCCCTTTGAGACAGTCTCACTGTC-3´ | EMSA  Mutagenesis |
| SRY2WTFOR  SRY2WTREV | 5’-GACAGTGAGACTGTCTCAAA**AAA**CAA**AAA**CAAAAACA**AAA**AAACATGGGCTGGGCGCAG-3’ 5´-CTGCGCCCAGCCCATGTTTTTTTGTTTTTGTTTTTGTTTTTTGAGACAGTCTCACTGTC-3´ | EMSA  Mutagenesis |
| SRY1MUTFOR  SRY1MUTREV | 5’-CGAGACTCCATCTCAAAAAC**GGG**CAAAG**GG**ACA**CC**CAACAACAACAACAACAAAAACCAT-3’  5´-ATGGTTTTTGTTGTTGTTGTTGTTGGGTGTCCCTTTGCCCGTTTTTGAGATGGAGTCTCG-3´ | EMSA  Mutagenesis |
| SRY1WTFOR  SRY1WTREV | 5’ CGAGACTCCATCTCAAAAAC**AAA**CAAAG**AA**ACAAACAACAACAACAACAACAAAAACCAT-3’  5´ ATGGTTTTTGTTGTTGTTGTTGTTGTTTGTTTCTTTGTTTGTTTTTGAGATGGAGTCTCG-3´ | EMSA  Mutagenesis |
| SRY0MUTFOR  SRY0MUTREV | 5´-GTTCCCAGCACAGTCATGGATGGAAACTCAGTGCCTGAAAAGGC-3´  5´-GCCTTTTCAGGCACTGAGTTTCCATCCATGACTGTGCTGGGAAC-3´ | Mutagenesis |
| SRY F | 5´-CAC*GGATCC* ATG CAA TCA TAT GCT TCT GC 3′ | Cloning |
| SRY R | 5´-TTT*GAATTC* CTA CAG CTT TGT CCA GTG G -3′ | Cloning |

**S1 Table. Primers used for generating *CATSPER1* promoter deleted and mutated constructs**
